# An overview of GeoAI applications in health and healthcare

**DOI:** 10.1186/s12942-019-0171-2

**Published:** 2019-05-02

**Authors:** Maged N. Kamel Boulos, Guochao Peng, Trang VoPham

**Affiliations:** 1 School of Information Management, Sun Yat-sen University, East Campus, Guangzhou, 510006 Guangdong China; 2 Channing Division of Network Medicine, Department of Medicine, Brigham and Women’s Hospital and Harvard Medical School, 181 Longwood Ave, Boston, MA 02115 USA

**Keywords:** Artificial intelligence, Big data, Deep learning, GeoAI, GIS, Geomedicine, Health, Healthcare, Internet of Things, Machine learning, Precision medicine, Public health, Smart healthy cities

## Abstract

The moulding together of artificial intelligence (AI) and the geographic/geographic information systems (GIS) dimension creates GeoAI. There is an emerging role for GeoAI in health and healthcare, as location is an integral part of both population and individual health. This article provides an overview of GeoAI technologies (methods, tools and software), and their current and potential applications in several disciplines within public health, precision medicine, and Internet of Things-powered smart healthy cities. The potential challenges currently facing GeoAI research and applications in health and healthcare are also briefly discussed.

## Introduction

Artificial intelligence (AI), such as methods in machine learning, has been increasingly used in health and healthcare, particularly with the rise of high-performance and cloud computing capabilities [1, 2]. Health intelligence refers to the specific application of AI and data science methods and tools to provide accurate, efficient, and productive insights into healthcare and medicine [3]. Health intelligence applications have included social media analytics for syndromic surveillance [4], predictive modelling to identify populations at high risk for disease [5], mobile health for healthcare delivery [6], and medical imaging interpretation [3, 7].

Broadly, health intelligence can be characterised as applications of AI to improve health at the population level and at the individual level. Population-level applications include those aimed at promoting public health such as through the disciplines of environmental health, epidemiology, genetics, social and behavioural sciences, and infectious diseases. In contrast, individual-level applications can be geared towards precision medicine, or disease management that considers individual variability in genetics, environment, and lifestyle [3, 8]. Regardless of the target scale of the population or individual, location or place is an important consideration in health intelligence as it can play a significant role in health. The locations in which we live, work, and spend our time are associated with factors, including but not limited to the built environment, environmental exposures, and social determinants, that may impact our health. Incorporating location-based information can allow us to better understand risk factors for disease and identify novel targets for prevention efforts.

Spatial science offers tools and technologies that enable us to understand, analyse, and visualize real-world phenomena according to their locations [2]. Geospatial artificial intelligence (GeoAI) combines methods in spatial science (e.g., geographic information systems or GIS), AI, data mining, and high-performance computing to extract meaningful knowledge from spatial big data [2, 9]. GeoAI represents a focused domain within health intelligence that incorporates location to derive actionable information that can be used to improve human health. A common theme across GeoAI applications at the population and individual level is the use of novel sources of spatial big data, such as social media, electronic health records, satellite remote sensing, and personal sensors, to advance the science of public health (especially in the context of ‘smart healthy cities’) and potentially precision medicine, creating new opportunities to more comprehensively answer questions typically tackled in these fields as well as unique opportunities to answer new, emerging questions.

The purpose of this editorial is to provide an overview of GeoAI technologies (methods and tools, and software), and current and emerging/potential applications of GeoAI in several disciplines within public health and precision medicine, as well as IoT (Internet of Things)-powered smart healthy cities. Smart healthy cities gather, generate and consume large amounts of big health and environmental data. GeoAI can play a key role in making sense of these data through intelligent, location-based big data analytics.

## GeoAI technologies

Geospatial data refers to data containing a geographic component that identifies locations (e.g., coordinates, addresses, and postcodes) or indicates geographically referenced features and conditions, such as the population of a district, seasonal weather of a region, number of vehicles passing a highway intersection, and geo-tagged social media data [10]. National and local governments were historically the main provider of geospatial data, but it is becoming common for geospatial data to be acquired and generated by commercial enterprises, academic researchers, and non-profit organisations [10]. More recently, IoT sensors and devices deployed in modern cities represent novel and alternative sources of generating geo-tagged big data [11]. It is therefore meaningful and imperative to apply robust GeoAI technologies to process, analyse, and make sense of such increasing amounts of spatial big data in real time [11].

Machine learning, deep learning and data mining are essential methods forming the foundation of GeoAI. In particular, machine learning includes AI methods and algorithms for computers to obtain knowledge by iteratively extracting and learning from patterns hidden in raw data [12]. Deep learning is widely perceived as a cutting-edge type of machine learning that allows computers to simulate brain function to understand complex concepts in the real world more effectively [12]. Data mining techniques were developed as part of machine learning to explore new patterns from large datasets and to make appropriate recommendations (e.g., recommender systems in e-commerce sites) [12]. GeoAI tools and applications aim to utilise all of these methods as relevant to obtain valuable information and knowledge from spatial big data for specific analytical needs [12]. Such GeoAI tools and software have been developed and applied in different domains and contexts, including for the military, commercial businesses, and public and civic sectors.

For example, Situational Awareness Geospatially Enabled (SAGE) is a military application and a GeoAI tool developed by the US Army Engineer Research and Development Centre [13]. The SAGE tool utilises and analyses four types of geospatial data (e.g., elevation, terrain categorization, road names, and map imagery) to create tactical information for understanding the operational environment and supporting military decision making (e.g., suggesting feasible and safe routes for commanders to send troops) [13]. In addition, the Ship Rider program launched by the US National Geospatial-Intelligence Agency (NGA) has been developing, promoting and applying tools to deliver on-demand GeoAI outputs to the US Navy [14]. The program incorporates mapping, charting, geodesy and imagery into geospatial intelligence analysis and allows the Navy to visualise and obtain critical spatial insights at sea [15].

In the commercial sector, the combination of business data, operational data and geospatial data with analytics and mapping visualisations has been suggested to provide valuable insights across many industries [16]. There are several industrial use cases that have demonstrated how GeoAI could enhance existing business intelligence software and improve business efficiency and competitiveness. For example, GeoAI tools (e.g., SAS Visual Analytics) were advantageous in sales forecasting, consumer demand prediction, and marketing analysis, enabling sales managers to identify locations of customers to provide the highest profit gains [17]. Beyond sales and marketing, GeoAI tools for visual analytics can add the “where” component to optimise other business processes in the product lifecycle, including manufacturing, assembling, logistics and distribution [16]. Based on deep geospatial insights, business managers can improve decision making related to manufacturing, storage and distribution plans, ensuring the production and delivery of end-products to customers using the most economical routes in the shortest amount of time [18].

As the primary provider of geospatial data since 1990s [10], the public and civic sector has pioneered the application of GIS and more recently GeoAI technologies. In particular, the US government has been utilising geospatial intelligence tools for generating city development plans, mapping foreclosure and crime rates, and monitoring and responding to natural hazards such as floods, wildfires and hurricanes in real-time [10]. Further, less developed countries, such as Ecuador, have been using geospatial intelligence for health planning and determining regional distribution of health services [19]. The World Health Organization (WHO) has been applying geospatial mapping tools for global applications, including for the Ebola virus disease outbreak in 2014, to monitor and respond to the emergence of new disease cases in different countries over time [20].

## GeoAI for public health

Public health seeks to promote health and prevent disease at the population level, involving multiple specialised disciplines that aim to understand and/or intervene on different aspects of population health. Several examples of recent and emerging GeoAI applications in the disciplines of environmental health, epidemiology, genetics, social and behavioural sciences, and infectious diseases are provided below.

In environmental health, GeoAI has been used to conduct accurate and highly resolved modelling of environmental exposures, including measuring exposures that have historically been difficult to capture [2]. For example, GeoAI methods are being applied to capture features of the built environment (i.e., urban green space or natural environments) [21]. To address current limitations regarding the lack of green space measurements at the highly granular street scale, one recent study calculated Green View Index (GVI) measures from Google Street View panorama images in Portland, Oregon, US and compared GVI values to conventional green space measures such as normalised difference vegetation index (NDVI), distance to parks, and neighbourhood socioeconomic status [21]. Correlations between GVI and other green space measures were low, suggesting that GVI captures unique information not otherwise ascertained using existing methods. Future research building on this work includes developing machine learning approaches to identify specific green space features (e.g., trees) and complex characterizations of streetscapes [21]. In another study designed to address the paucity of building maps in developing countries for sustainability goals related to disaster relief and poverty reduction, deep learning (convolutional neural networks or CNNs) were applied to WorldView-2 satellite remote sensing images and volunteered geographic information (VGI) to automate map generation for buildings in Nigeria [22]. Future research could scale these approaches to examine larger study areas to conduct population-based research and to incorporate more time points to produce a high spatiotemporal resolution characterization of the built environment.

Machine learning has experienced an increased presence in air pollution exposure modelling, allowing for methodologic advantages such as modelling nonlinear associations and the integration of multiple spatial big data sources to improve predictive performance [23]. For example, a neural network was used to model daily particulate matter < 2.5 microns in diameter (PM_2.5_) levels in the US using multiple predictors including satellite-based aerosol optical depth (AOD) from the Moderate Resolution Imaging Spectroradiometer (MODIS) aboard the Earth Observing System satellite [24]. Random forest models have also been used to estimate daily PM_2.5_ concentrations [25, 26]. A geographically-weighted gradient boosting machine (GW-GBM) algorithm was used to model PM_2.5_ exposures in China, accounting for spatial non-stationarity in associations between predictors and PM_2.5_ using spatial smoothing kernels [2, 27].

Beyond satellite remote sensing, mobile air pollution sensors is another novel source of spatial big data that has been used to improve air pollution exposure modelling [28]. Urban air pollution concentrations exhibit high variability over short distances due to unevenly distributed emission sources, dilution, and physicochemical transformations [28]. Air pollution sensors were integrated with Google Street View vehicles to sample every street in Oakland, California, US for air pollution mapping of NO, NO_2_, and black carbon at a 30 m spatial resolution [28]. Spatial data mining techniques were used to explore determinants of the spatial patterns in the measured levels of air pollution [28]. Further, personal exposure to air pollution has been measured using wearable devices (i.e., wearables), providing continuous measurements through portable air pollution sensors attached to wristbands, belts, and backpacks [29]. For example, a personal sensor for ultrafine particles (UFP; < 100 nm in diameter) measured pollutant levels among adolescents in Cincinnati, Ohio, US [29]. The UFP sensor also incorporated a Global Positioning System (GPS) receiver, which appended geolocations to corresponding UFP measurements [29]. High-dimensional spatial big data gathered by wearables can be processed and analysed using data science methods and incorporated into epidemiological studies studying disease risk as described below.

In epidemiology, GeoAI has been used to describe and analyse the spatial distribution of diseases and to study the effect of location-based factors on disease outcomes. For example, to facilitate hypothesis generation related to the aetiology of preterm births, machine learning (K-means clustering) was used to determine spatiotemporal patterns of gestational age at delivery for 145 million births in over 3000 US counties from 1971 to 2008 using National Centre for Health Statistics Natality Files [30]. In another study in the Ivory Coast of Africa, researchers aimed to better understand determinants of human immunodeficiency virus (HIV) prevalence using machine learning (support vector regression) to extract mobility and connectivity data from georeferenced mobile phone data [31]. These extracted features were analysed in relation to HIV prevalence rates at the department level, where study authors found that factors, such as the spatial area covered by the phone user and overall migrations, were associated with HIV prevalence [31].

Environmental health also has close ties with epidemiology, as measured and/or modelled environmental exposures can be used for exposure assessment in study populations as part of environmental epidemiological studies. For example, a recent study used CNNs on Google Maps Static images and Google Places (of interest) to extract natural and modified elements of the built environment (e.g., buildings, crosswalks, street greenness) to study in relation to Census tract-level obesity prevalence in the US from the Centres for Disease Control and Prevention (CDC) 500 Cities project [32]. This research can inform neighbourhood-level interventions to increase physical activity and access to healthy food outlets to address the obesity epidemic. Another epidemiological study linked deep learning-based air pollution exposure models for PM_2.5_ and ozone with ZIP Codes from Medicare big data to examine their associations with mortality risk in the US [33]. These examples illustrate the numerous opportunities for GeoAI to model environmental exposures that can be subsequently linked with various data sources with information on disease outcomes (and potential confounders) to conduct epidemiological research.

Furthermore, future epidemiological research could harness emerging sources of spatial big data to examine research questions regarding disease aetiology, potentially providing new insights into novel risk factors. For example, personal sensing collects data using the sensors embedded in mobile phones as well as through wearables such as Fitbits [34]. Spatial energetics is a field that focuses on collecting high spatiotemporal resolution data on location and time-matched energetics from GPS, accelerometery, and GIS to identify spatial-based factors that may be associated with physical inactivity and obesity [35]. GeoAI could be used to process and analyse these location-based data to determine what types of activities at certain times and exposures at specific locations for different types of people are relevant to health outcomes. Other novel spatial big data sources include information from ride sharing services such as Uber and Lyft. There are approximately 5.5 million Uber rides and 1 million Lyft rides completed each day [36]. Location is a key aspect of ride sharing as it relates to pick-ups and drop-offs; this information can be used to address epidemiological research questions related to injury, for example. Some research suggests that areas characterised by higher usage of ride sharing may be associated with lower incidence of traffic-related accidents, although improved study designs as well as data (i.e., analysing ride sharing rates rather than dates of rollout) are needed [37]. Further, food delivery as part of ride sharing services (e.g., Uber Eats) may also provide interesting insights into its potential role in promoting sedentary behaviours and childhood obesity [38].

In genetics, deep learning has been applied to studying fields such as functional genomics (e.g., predicting the sequence specificity of DNA- and RNA-binding proteins) [39]. Gene-environment interaction (GxE) studies represent an opportunity to apply GeoAI towards examining the intersection of genetics and the environment (through location-based information) on health. GxE studies provide insights into understanding disease, from disease biology to identifying genetic subgroups with higher exposure-specific disease risk [40]. A current limitation of GxE research includes the complexity of measuring environmental exposures such as accounting for the appropriate temporality of environmental exposures, measurement error, and limited environmental exposure variability [40]. Many of the aforementioned novel spatial big data sources can be viewed as potential ways through which to measure the exposome, or the totality of human environmental (i.e., non-genetic) exposures from conception onwards [41]. For example, location-based measures from remote sensing, smartphone apps, and personal exposure sensors could be incorporated into GxE studies for exposure assessment during time periods relevant to the disease of interest.

In social and behavioural sciences, GeoAI has been used to help identify social and behavioural determinants of health as well as to conduct interventions using locational information. Electronic health records (EHRs) are a valuable longitudinal population-based big data source. EHRs allow for the linkage of spatial data to geographic variables such as ZIP Codes as patient addresses are routinely checked and updated for billing purposes [42]. For example, an analysis of EHRs from the Duke University Health System and Lincoln Community Health Centre in the US used machine learning (random survival forests) to determine if neighbourhood-level socioeconomic status (SES) improves risk prediction of health outcomes such as emergency department visits and inpatient visits [43]. US Census Bureau American Community Survey data were used to determine neighbourhood-level SES by calculating the Agency for Healthcare Research and Quality SES index at the Census tract level. Another study used machine learning to implement an intervention for depression [44]. A mobile phone app was developed using machine learning to predict patient mood, emotions, cognitive/motivational states, activities, environmental context, and social context based on over 30 phone sensors such as GPS [44]. Deep learning (neural networks) have also been used to identify social determinants (i.e., income, wealth, education) that predict health outcomes including systolic blood pressure, body mass index, waist circumference, and telomere length in the US-based Health and Retirement Study [45]. This approach could incorporate location-based measures, including area-level SES, as potential social determinants to investigate in relation to health.

Dating apps, such as Tinder, are a feature of modern dating that could be a novel data source to answer health-related research questions. There are over 4 million paying users on Tinder [46]. Tinder is an example of a proximity-based dating application that sets up a specific radius using geolocation technology to allow its users to find potential partners located within their vicinity [47]. These georeferenced data could be used to examine associations between geographic variation in dating app use and dating violence and abuse [48] or depression [49].

GeoAI has been used in infectious disease research for modelling or prediction of disease occurrence and for disease surveillance. Deep learning recurrent neural networks (RNNs) were used for real-time influenza forecasting at regional and city spatial scales in the US using spatial big data on Google Flu Trends (weekly estimates for different cities) and climate (e.g., precipitation, temperature, sun exposure) from the National Climatic Data Centre [50]. Another application utilised geotagged tweets from Twitter and the CDC influenza-like illness (ILI) dataset to predict real-time regional ILI in the US using an artificial neural network (ANN) optimised by an artificial tree algorithm [51]. The geotagged tweets were based on the location in the profile of the Twitter user who tweeted the message, the location where the user sent the tweet and enabled their geographical location tracking in the Twitter App, or the location mentioned in the content of the tweets. In another study, an ensemble machine learning approach was used to estimate state-level influenza activity in the US, combining a self-correcting statistical method with influenza-related Google Trends, cloud-based athenahealth EHRs, and historical flu trends, as well as a network-based approach leveraging spatiotemporal trends in historical influenza activity [52].

Machine learning (support vector regression) was used for dengue fever forecasting in China using data on climate, weekly dengue fever cases, and Baidu search queries [53]. Validation showed that epidemics during the previous 12 weeks and the peak of the 2014 large outbreak were accurately forecasted. Another study developed a machine learning model called FINDER to detect foodborne illnesses using anonymous and aggregated Google web search and location data, estimating the fraction of people who visited a particular restaurant and who subsequently searched for terms indicative of food poisoning (to identify potentially unsafe restaurants) [54]. This information was used to focus restaurant inspections, showing that restaurants identified by FINDER were more likely to be deemed unsafe during the inspection compared to existing methods. A real-time syndromic surveillance system was developed to detect disease outbreaks earlier, where deep learning classified health-related geotagged Tweets (e.g., tweets with news sources with location as part of the news article) and allowed for the geovisualisation of health symptoms [4]. This system demonstrated an ability to detect ILI symptoms, which were confirmed from the CDC Morbidity and Mortality Weekly Reports (MMWR). Future research to improve on this surveillance system will incorporate disease-specific information (e.g., mode of transmission) to enhance disease forecasting accuracy.

## GeoAI for precision medicine

The practice of medicine involves making decisions based on obtaining as much information about a patient’s health as possible [55]. Precision medicine is an effort to tailor prevention and treatment strategies through considering individual variability in genetics, environment, and lifestyle [8]. Applications of AI in precision medicine have included using machine learning for prediction of patient diagnoses and outcomes [56]. Opportunities to incorporate GeoAI can be found in emerging research initiatives focused on the integration of mobile health (mHealth) in precision medicine. mHealth is the application of mobile technologies (e.g., phones, tablets) to support and enhance the performance of healthcare and public health practice [57]. One research focus of the National Institutes of Health (NIH) Big Data Centre of Excellence on Mobile Sensor Data-to-Knowledge (MD2 K) is the design of sensor-triggered mHealth interventions, which could integrate information on sensor-based environmental exposures such as light, noise, chemicals, etc. to improve the temporal precision of mHealth-based precision medicine [58]. For example, MD2 K developed mCerebrum, a configurable smartphone software platform supporting reliable high-frequency data collection from mobile and wearable sensors and real-time processing of these data for sensor-triggered, just-in-time adaptive interventions [58]. Another example of mHealth incorporating location-based exposures is AirRater, which is an integrated online platform that combines environmental monitoring, symptom surveillance, and notifications of changing environmental conditions through a smartphone app [59]. AirRater modelled PM_2.5_, pollen, and meteorological variables using high temporal resolution environmental monitoring data and spatial interpolation methods (e.g., kriging). Participants, most of whom reported having either asthma or allergic rhinitis, created saved locations via the app and were sent notifications when these saved locations experienced elevated PM_2.5_ or pollen levels. AirRater users reported developing a better understanding of how environmental conditions affect their health, which sometimes prompted action such as their timely use of medications.

Another potential application of GeoAI for precision medicine is through geomedicine, a term that has been used to describe considering the importance of a patient’s place history in disease diagnosis and treatment [60]. Clinicians could be delivered information on a patient’s environmental exposures, which could help clinicians identify environmental factors that may influence a patient’s health. Such endeavours may include clinicians (having access to patient residential histories) providing patients with information regarding potential ambient exposures to environmental risk factors based on where they live and work—and these environmental exposures could be derived via GeoAI technologies. However, barriers to such applications include clinical acceptability, particularly as the translation of precision medicine into clinical care and health policy in general has lagged behind the pace of scientific discoveries [40]. Yet accelerations in this area may be likely, as the cost of sequencing the human genome has decreased substantially in recent years and more patients are expected to survey their own personal genomes (e.g., 23 and Me genetic testing) as a means to monitor and improve their personal health [61]. Potentially informed by GxE research, patients who discover genetic susceptibility to particular diseases, for example, may benefit from information regarding location-based environmental exposures that may be especially damaging to their own personal health [62].

A notable research endeavour in precision medicine is the NIH All of Us Research Program, which aims to collect data from one million or more people living in the US to accelerate research to improve health outcomes, advance the development of new disease treatments, and contribute to evidence-based research to develop more precise preventive care and medical treatments [63]. One area of focus is using mHealth technologies to correlate activity, physiological measures, and environmental exposures with health outcomes, which represents an opportunity to incorporate location-based exposures, and thus GeoAI technologies, into precision medicine research.

## GeoAI for IoT-powered smart healthy cities

IoT-powered smart cities rely heavily on the usage of sensors that can be embedded into buildings, roads, vehicles, devices, plants, animals and human bodies turning these physical objects into digitally connected “things” [11]. These IoT sensors deployed in cities lead to the generation of a huge amount of real-time data, which are often geo-tagged or geo-located. GeoAI is essential for processing and making sense of such geospatial and real-time big data, and support the smart vision of cities. For example, cities such as Barcelona [11], New York [64] and Dublin [65] have been adopting smart/connected bins with wireless sensors to detect and monitor trash levels in real time. With the support of GeoAI, trash collectors will then be informed and constantly updated regarding optimal routes for garbage collection in locations that require attention. In this scenario, the combination of IoT big data and GeoAI allows cities to minimise waste management costs and effort with improved efficiency and smartness [11].

In light of this discussion, smart city initiatives include inherent components for a healthier environment, referring to the concept of smart healthy cities that aim to improve the quality of city lives and enhance wellbeing of citizens [11]. The GeoAI applications in public health and precision medicine discussed above can all contribute to make smart healthy cities a reality. The fundamental aim should be to expand and integrate GeoAI endeavours in public health and precision medicine with other IoT infrastructures deployed in smart cities to facilitate large-scale effects at the population level.

For example, in Barcelona, a city-wide network of IoT sensors and connected devices (e.g., smart bins, smart streetlights with embedded air quality monitoring sensors, smart parking spots, and high-resolution cameras), provides valuable real-time data on noise, air quality and other types of environmental pollutants as well as the flow of citizens and traffic conditions, covering diverse locations across the city [11]. The comprehensive analysis of these geo-tagged IoT data can allow local authorities to identify the most crowded and polluted areas in the city at different time points, enabling instantaneous decision making (e.g., actively changing driving speed limits in busy intersections) to ease traffic pressure and reduce environmental burden. In addition, the integration of health big data (e.g., EHRs, mHealth and wearable sensing data) with city IoT infrastructure and GeoAI tools can allow local authorities and policy makers to enhance city development plans to distribute and improve public services related to health and transportation (e.g., targeted development of hospitals and care centres proximate to local transportation in areas with a large population requiring caring needs). In addition, when responding to city emergencies and disasters, GeoAI tools can be used to process and analyse geo-tagged IoT datasets, generating city maps for navigating the affected areas and obtaining contextual and real-time information (e.g., traffic and injured/patient conditions) for emergency responders [66].

The integration of health big data and IoT sensing data could maximise the utility of GeoAI in realising the vision of smart healthy cities. However, such integration may not be easily achievable in practice. For example, China has initiated over 700 smart city projects in more than 500 cities since 2012. This endeavour led to the emergence of hundreds of smart city apps and services on health and transportation [67]. While many Chinese cities have expressed an interest in integrating their various local smart city apps into one single platform, these attempts remain in preliminary stages [67]. The absence of adequate standards and protocols for communication, integration, interoperability and control is a major barrier hindering seamless integration of IoT systems and applications in smart cities [11, 68], which together with data privacy and methodological issues discussed below, can influence the development and usage of GeoAI tools in IoT-powered smart healthy cities.

## Potential challenges

The US NIH is expected to fund more research into mobile imaging, pervasive sensing, social media, and location tracking in the future [69]. As massive amounts of data continue to be captured and collected, issues related to data privacy are paramount. Ethical frameworks are also required to appropriately inform study participants of risks and to protect patient privacy. Standards for securing and sharing research data collected by commercial devices and apps should also be addressed.

A methodological challenge is the lack of labelled training data for AI algorithms. In particular, supervised learning involves predicting the label or response of each data point using a set of labelled training examples [39]. Training data are important to accurately identify geographic features from input data. There are initiatives in place to facilitate this process, including Google’s human labelling service that allows human operators to label images. Importantly, as AI methods becomes more pervasive in clinical research, the role of subject matter expertise becomes imperative to avoid the uninformed use of big data as part of AI algorithms to produce results [2, 70]. Domain expertise is needed to avoid erroneous discoveries and to properly understand the relationships being modelled. Although GeoAI is valuable for discovery and hypothesis generation, there is a significant need for well-designed studies and use of appropriate data to confirm any findings from this research [70].

## Conclusions

There is an emerging role for GeoAI in health and healthcare as location is an integral part of both population and individual health. Novel sources of spatial big data, including social media, satellite remote sensing, and personal sensing, are being (and could be) analysed to answer research questions in more nuanced ways as part of a variety of disciplines including environmental health, epidemiology, genetics, social and behavioural sciences, and infectious diseases. GeoAI has been used to model and capture the environment around us, linking locations in which we live, work, and spend our time to these exposures (whether it be environmental, social, etc.) to explore their potential role in influencing health outcomes. GeoAI has also led to research for hypothesis generation, conducting new data linkages, and predicting disease occurrence. There are currently numerous population-level GeoAI applications for public health and IoT-powered smart healthy cities, and there are emerging opportunities for integration of GeoAI and location-based information into precision medicine such as via mHealth for interventions. Future research can expand on current GeoAI applications, such as modelling location-based features that have not been previously captured at a high spatiotemporal resolution, or analytics for newly emerging spatial big data source, to unlock new areas of research and advance our understanding of human health.
